# Genome-Wide Association Study of Antiphospholipid Antibodies

**DOI:** 10.1155/2013/761046

**Published:** 2013-02-24

**Authors:** M. Ilyas Kamboh, Xingbin Wang, Amy H. Kao, Michael M. Barmada, Ann Clarke, Rosalind Ramsey-Goldman, Susan Manzi, F. Yesim Demirci

**Affiliations:** ^1^Department of Human Genetics, Graduate School of Public Health, University of Pittsburgh, Pittsburgh, PA 15261, USA; ^2^Division of Rheumatology, Department of Medicine, West Penn Allegheny Health System, Pittsburgh, PA 15212, USA; ^3^Divisions of Clinical Immunology/Allergy, and Clinical Epidemiology, Department of Medicine, McGill University, Montreal, QC, Canada H3A 1A1; ^4^Division of Rheumatology, Feinberg School of Medicine, Northwestern University, Chicago, IL 60611, USA

## Abstract

*Background*. The persistent presence of antiphospholipid antibodies (APA) may lead to the development of primary or secondary antiphospholipid syndrome. Although the genetic basis of APA has been suggested, the identity of the underlying genes is largely unknown. In this study, we have performed a genome-wide association study (GWAS) in an effort to identify susceptibility loci/genes for three main APA: anticardiolipin antibodies (ACL), lupus anticoagulant (LAC), and anti-**β**_2_ glycoprotein I antibodies (anti-**β**_2_GPI). *Methods*. DNA samples were genotyped using the Affymetrix 6.0 array containing 906,600 single-nucleotide polymorphisms (SNPs). Association of SNPs with the antibody status (positive/negative) was tested using logistic regression under the additive model. *Results*. We have identified a number of suggestive novel loci with *P* < *E* − 05. Although they do not meet the conservative threshold of genome-wide significance, many of the suggestive loci are potential candidates for the production of APA. We have replicated the previously reported associations of HLA genes and *APOH* with APA but these were not the top loci. *Conclusions*. We have identified a number of suggestive novel loci for APA that will stimulate follow-up studies in independent and larger samples to replicate our findings.

## 1. Introduction

Antiphospholipid antibodies (APA) are a heterogeneous group of antibodies that are detected in a variety of conditions, including primary antiphospholipid syndrome (APS) and systemic lupus erythematosus (SLE) [1]. The term antiphospholipid antibodies is a misnomer as APA present in autoimmune disease, like SLE, do not bind to phospholipids but recognize phospholipid-binding proteins [2]. Patients with persistent APA who develop pregnancy complications or thrombosis are considered to have primary APS and those who develop these complications in the presence of autoimmune disease are classified having secondary APS. Since the definition of APS is not limited to a single APA assay, it is required to measure more than one APA. Indeed, currently recognized laboratory criteria for APS include having one or more of three APA, including anticardiolipin antibodies (ACL), lupus anticoagulant (LAC), or anti-*β*_2_ glycoprotein I antibodies (anti-*β*_2_GPI) in conjunction with the presence of thrombosis or pregnancy loss [3]. 

Although the genetic basis of APA [4] and APS [5] has been suggested, the underlying genetic factors have not been clearly established. Understanding the genetic bases of various APA may help to delineate the mechanisms for APS. The objective of this study was to perform a genome-wide association study (GWAS) in an effort to identify loci/genes for the three main APA, namely, ACL, LAC, and anti-*β*_2_GPI.

## 2. Subjects and Methods 

### 2.1. Subjects

A subset of individuals from our larger GWAS of SLE (unpublished data) that had the ACL (*n* = 670), LAC (*n* = 708), and anti-*β*_2_GPI (*n* = 496) measurements available were used in this study. All individuals were women of European ancestry. The study participants included both SLE cases and controls and their characteristics are given in Table 1. Our controls were apparently healthy individuals that were recruited from blood bank. We measured APA in our controls but they were not characterized for primary APS due to our study design that is focused on identifying genes for SLE and APA. Furthermore, there were only 28 individuals with APS, and this small number was not considered to be appropriate for a GWAS analysis. All subjects provided written informed consent and the study was approved by the Institutional Review Board.

### 2.2. Antiphospholipid Antibodies

 The presence of ACL (IgG > 15 GPL units, IgM > 10 MPL units, IncStar, Stillwater, MN, USA), LAC (partial thromboplastin time or Russell's viper venon time with mix) and anti-*β*_2_GPI (QUANTA Lite *β*_2_GPI screen, INOVA Diagnostics, Inc. San Diego, CA, USA) was tested in sera or plasma obtained from the study subjects. The three APA (ACL, LAC, and anti-*β*_2_GPI) were classified into antibody-positive and antibody-negative groups based on manufacturer's protocols. 

### 2.3. Genotyping and Quality Control (QC)

DNA samples were genotyped using the Affymetrix Genome-Wide Human SNP Array 6.0 containing 906,600 SNPs at Expression Analysis, Durham, NC, USA. All samples used in this study passed strict quality control measurements in our larger GWAS. Exclusion criteria included samples with poor performance (<95% average call rate across the array), poorly performing markers (44,592 with <95% call rate across all samples genotyped), and markers with significant deviation from Hardy-Weinberg equilibrium (*P* ≤ 1*E* − 06) and with low minor allele frequency (MAF <0.01). Population stratification analysis was conducted using a multidimensional scaling method implemented in PLINK. SNPs falling within the genomic regions with abnormal linkage disequilibrium patterns and structural variations (hg18; chr2: 130–140 Mb, chr6: 24–36 Mb, chr8: 8–12 Mb, chr11: 42–58 Mb, and chr17: 40–43 Mb) were excluded from the principal component (PC) analysis but were included in subsequent association analysis. First 4 components were determined to be relevant for the determination of population origin based on visual examination of PC plots and were used as covariates in the association statistics. 

### 2.4. Association Analysis

 The three APA (ACL, LAC, and anti-*β*_2_GPI) were classified into antibody-positive and antibody-negative groups based on manufacturer's protocols. Association of SNPs with the antibody status was tested using logistic regression under the additive model. Considering the effect of SNPs on the antibody status may be confounded by the disease status (SLE) and other demographic variables (age, BMI, smoking), we used the stepwise regression method to select the most parsimonious set of covariates for each dependent variable. The analysis for each antibody was adjusted for the disease status (SLE) and the first four principal components. In addition, the ACL and LAC analyses were adjusted for smoking and BMI, respectively. *R* and/or PLINK statistical software programs were used for all analyses performed for this study. 

## 3. Results

### 3.1. Quantile-Quantile Plots of the GWAS Data

**Figure 1:**
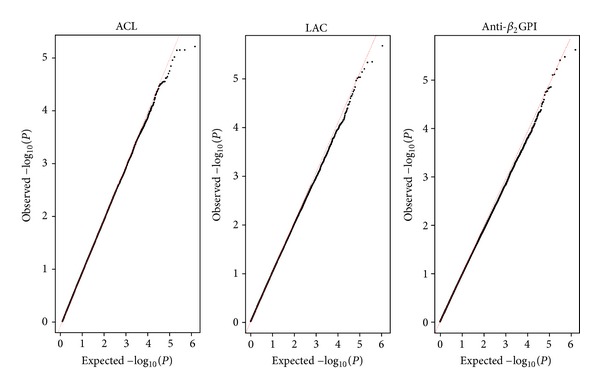
Quantile-quantile plots of the observed versus the expected *P* values for ACL, LAC, and Anti-*β*_2_GPI.

The genome-wide association analysis was performed on 670 individuals with ACL, 708 individuals with LAC and 496 individuals with anti-*β*_2_GPI (Table 1) who were genotyped using the Affymetrix Genome-Wide Human SNP Array 6.0.  Figure 1 shows the quantile-quantile plots for comparisons of observed and expected *P* values distribution for ACL, LAC, and anti-*β*_2_GPI. For all three APA, the distribution of observed *P* values conformed to the null distribution until the tail of the distribution where it deviated, indicating no evidence of significant population stratification but evidence of genetic association.

### 3.2. Association with Anticardiolipin Antibodies (ACL)

**Figure 2:**
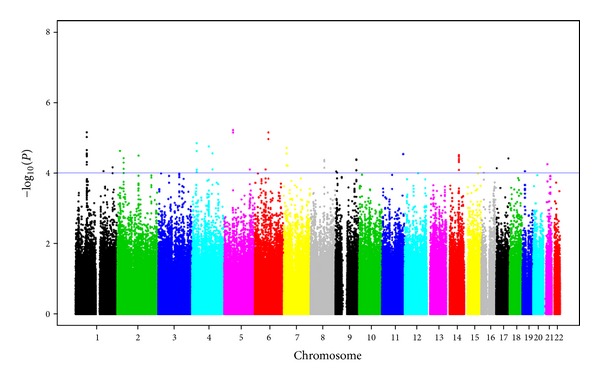
Manhattan plot showing the genome-wide association *P* values with anticardiolipin antibodies (ACL). Blue line indicates *P* = 1*E* − 04.

Figure 2 shows the genome-wide *P* values for ACL in a Manhattan plot and the top loci with *P* < 1*E* − 04 are presented in Table 2. Three top SNPs with *P* < 1*E* − 05 were observed. The most significant SNP, rs6889746 (*P* = 6.02*E* − 06), was located upstream of *PELO* (Pelota homolog) on chromosome 5q11.2. The next top SNP, rs6681460 (*P* = 6.98*E* − 06), was present in *SGIP1* (SH3-domain GRB2-like-intercation protein1) on chromosome 1p31.3. There was a total of 28 SNPs in this region with *P* < 1*E* − 03. The next top SNP, rs12204683 (*P* = 7.02*E* − 06), resided downstream of *LCA5* on chromosome 6q14.1.

### 3.3. Association with Lupus Anticoagulant (LAC)

**Figure 3:**
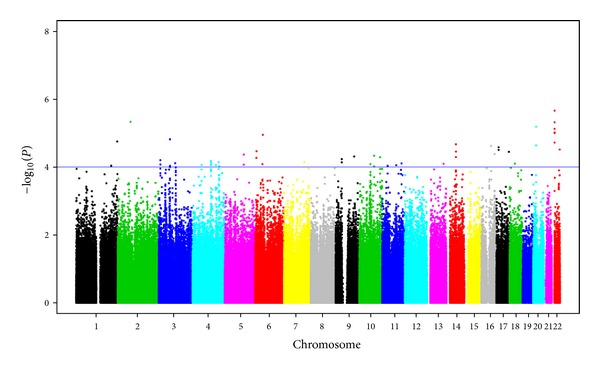
Manhattan plot showing the genome-wide association *P* values with lupus anticoagulant (LAC). Blue line indicates *P* = 1*E* − 04.

The Manhattan plot for LAC is shown in Figure 3 and the top hits with *P* < 1*E* − 04 are given in Table 3. The most significant SNP, rs1978968, was observed in *MICAL3 *on chromosome 22q11.21 (*P* = 2.21*E* − 06) and there were additional 7 significant SNPs in this region with *P* < 1*E* − 03. The next significant SNP was observed on chromosome 2p12 in *FAM176A* (rs17011455, *P* = 4.70*E* − 06). However, no other SNP with *P* < 1*E* − 03 was observed in this region. The third significant SNP, rs17791782, was observed in *DSTN* on chromosome 20p12.1 (*P* = 6.54*E* − 06).

### 3.4. Association with Anti-*β*_2_ Glycoprotein I Antibodies (Anti-*β*_2_GPI)

**Figure 4:**
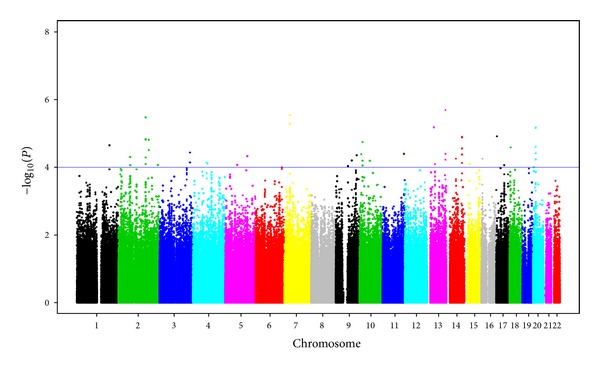
Manhattan plot showing the genome-wide association *P* values with anti-*β*_2_ glycoprotein I antibodies (Anti-*β*_2_GPI). Blue line indicates *P* = 1*E* − 04.

Five loci on four chromosomes were observed at *P* < 1*E* − 05 for association with anti-*β*_2_GPI (Figure 4, Table 4). The top SNP (rs10492418) at *P* = 2.05*E* − 06 was observed on chromosome 13q33.3 in *MYO16*. This chromosome also harbors another locus for anti-*β*_2_GPI at 13q14.11 (rs9315762, *P* = 6.68*E* − 06), near a region expressing long intergenic nonprotein coding RNAs. The second most significant SNP, rs11975235, was observed in *PDE1C* on chromosome 7p14.3 (*P* = 2.88*E* − 06). The third most significant SNP was observed upstream of *TANK* on chromosome 2q24.2 (rs2357982, *P* = 3.38*E* − 06) that also harbored 12 additional significant SNPs with *P* < 1*E* − 03.

### 3.5. Association with Presence of Two or More Antibodies

 In addition to the single-antibody analyses described above, we also performed an association analysis between individuals who were positive for two or more antibodies (*n* = 100) versus individuals who were negative for all three antibodies (*n* = 227).  Table 5 shows the results of top loci with *P* < 1*E* − 04. Interestingly, five of these loci (*SESTD1*, *CACNB2*, *TANK*, *TMEM45B,* and *FMN1*) overlapped with those observed in the anti-*β*_2_GPI analysis (see Table 4) and two (*DSTN* and *BFSP1*) overlapped with those observed in the LAC analysis (see Table 3). Although the most significant locus, *DYNLRB2* (*P* = 1.44*E* − 06), was not among the top loci detected in any of the single-antibody analyses, the second most significant locus, *SESTD1* (*P* = 6.08*E* − 06), also showed association with anti-*β*_2_GPI. 

### 3.6. Association of Extended Major Histocompatibility Complex (xMHC) Region and Apolipoprotein H (APOH) with APA

 Previously, several studies have reported genetic association of the human leukocyte antigen (HLA) genes located at the MHC locus on chromosome 6p21 with the presence of APA [6]. Likewise, since *β*_2_GPI is the main target antigen for APA, genetic variation in its gene, *APOH*, is expected to be associated with the occurrence of APA. Although no SNPs from either the HLA genes or *APOH* were among the top GWAS SNPs with *P* < 1*E* − 04 (Tables 2–5), the xMHC region revealed 104, 191, and 108 significant SNPs (*P* < 0.05) to be associated with ACL, LAC, and anti-*β*_2_GPI, respectively.  Table 6 lists significant SNPs with *P* < 0.01 in the MHC region for the three APA examined. Most significant SNPs were observed in or near *HLA-DPB1*, *HLA-DPB2*, *HLA-DPA1*, *HLA-DQA1*, *HLA-DQA2,* and *HLA-DMA*. Noteworthy, some SNPs were associated with more than one APA. For example, among the SNPs located upstream of *HLA-DQA2*, rs9275765 and rs9275772 were associated with LAC (*P* = 7.86*E* − 04) and anti-*β*_2_GPI (*P* = 3.15*E* − 03), rs9275793 with LAC (*P* = 8.84*E* − 04) and anti-*β*_2_GPI (*P* = 3.10*E* − 03), and rs9276298 with LAC (*P* = 1.33*E* − 03) and anti-*β*_2_GPI (*P* = 5.23*E* − 03). Likewise, rs2395357 near *HLA-DPB2* showed association with ACL (*P* = 4.34*E* − 04) and LAC (*P* = 1.09*E* − 02) and rs11539216 in *HLA-DMA* with ACL (*P* = 9.96*E* − 04) and LAC (*P* = 9.29*E* − 03). Of the 21 QC-passed SNPs present in or near *APOH*, six revealed nominal associations with anti-*β*_2_GPI, and the Trp316Ser variant (rs1801690) was the most significant SNP (*P* = 3.12*E* − 03) (Table 7). Two additional SNPs also showed nominal associations with LAC (*P* = 0.026, 0.027).

## 4. Discussion 

The persistent presence of APA, such as ACL, LAC, or anti-*β*_2_GPI, may lead to the development of antiphospholipid syndrome (APS), which may occur alone (primary APS) or in the presence of an autoimmune disease (secondary APS). Although the genetic basis of APA and APS has been suggested [4, 5], the precise identity of the causative genes is largely unknown. Here we report the first GWAS focused on identifying the susceptibility loci/genes for the occurrence of three main APA, namely, ACL, LAC, and anti-*β*_2_GPI. 

Initially, we performed separate genome-wide analyses for the three APA because the antigen specificity of APA is highly heterogeneous and each APA may have different genetic determinants. This seems to be confirmed in our GWAS results where none of the top loci for the three APA overlapped (see Tables 2–4). However, a single-antibody analysis may include individuals who are positive for more than one antibody in the antibody-positive group or may include individuals in the antibody-negative group who are positive for another antibody, which might have an effect on the genetic association outcome. In order to address this potential problem, we performed an additional genome-wide analysis on individuals who were positive for two or more APA as they presumably would have a higher genetic load of APA susceptibility genes and compared them with those who were negative for all three APA tested. Noteworthy, seven of the top loci observed in the latter analysis overlapped with the top loci observed in the individual analyses of anti-*β*_2_GPI and LAC (see Table 5). Although none of the observed top loci in any analysis met the strict criteria for genome-wide level of significance (*P* < 5*E* − 08), we have identified a number of suggestive genomic regions with *P* < *E* − 05 that are worthy of follow-up studies in independent samples. They include loci harboring *DYNLRB2* (*P* = 1.44*E* − 06) and *SESTD1* (*P* = 6.08*E* − 06) for individuals positive for at least two APA;* PELO *(*P* = 6.02*E* − 06), *SGIP1* (*P* = 6.98*E* − 06), and *LCA5* (*P* = 7.02*E* − 06) for ACL; *MICAL3 *(*P* = 2.21*E* − 06), *FAM176A* (*P* = 4.70*E* − 06), and *DSTN *(*P* = 6.54*E* − 06) for LAC; and *MYO16 *(*P* = 2.05*E* − 06), *PDE1C *(*P* = 2.88*E* − 06), *TANK *(*P* = 3.38*E* − 06), *FLJ42392* (*P* = 6.68*E* − 06), and *MACROD2 *(*P* = 6.86*E* − 06) for anti-*β*_2_GPI. 

While many of these loci are of unknown function in antibody production, some of them harbor candidate genes known to be involved in immune response and thus may be relevant to the production of APA. For example,* DYNLRB2 *is involved in immune signaling and genetic variation in this gene is associated with tuberculosis susceptibility [7]. SESTD1 binds several phospholipid species [8] and may thus serve as an autoantigen for APA. TANK (TRAF family member-associated NFKB activator) is believed to be important in type 1 interferon production [9] and has been suggested to play a role in hepatitis B and C infections [10, 11]. The *MYO16* (myosin XVI) locus has recently been implicated in diabetic nephropathy [12–14]. Interestingly, the presence of APA or APS is a strong risk factor for nephropathy [15–17] and one study has suggested that anti-*β*_2_GPI may be protective against lupus nephritis and renal damage [18]. FAM176A (a.k.a *TMEM166*) has been implicated in autophagy and apoptosis [19], two mechanisms with suggested roles in autoimmunity [20, 21]. 

Before the GWAS era, the focus of genetic studies on APA was mainly on candidate genes, with a major emphasis on HLA genes located at the MHC locus and to some extent on *APOH*. Since none of our top hits included SNPs from either the HLA genes or *APOH*, we examined the extent of association signals in these genomic regions. Indeed, we found a number of promising significant SNPs near or in various HLA genes to be associated with ACL, LAC, and anti-*β*_2_GPI (see Table 6). Our findings are consistent with previous reports that also found multiple associations of HLA genes with these autoantibodies [6]. Previous findings regarding the association of *APOH* coding SNPs with APA have been inconsistent because of the conflicting reports [6, 22]. In our sample, we found six *APOH* SNPs to be associated with anti-*β*_2_GPI and the most significant SNP was rs1801690 (Trp316Ser) (see Table 7) that is located in the 5th domain of *β*_2_GPI affecting the phospholipid-binding site [23]. Another coding SNP in *APOH*, rs3176975 (Val247Leu), that has been reported to be associated with APS [22], showed only a modest trend for association in our sample (odds ratio 1.21; *P* = 0.286). The replication of previously reported HLA and *APOH* findings with similar association signals serve as positive controls for our GWAS. On the other hand, it also indicates that HLA and *APOH* are not among the top loci for APA and thus our focus should be on the identification and characterization of other genes that are more relevant to the production of APA.

In conclusion, to the best of our knowledge, this is the first GWAS that has attempted to delineate the genetic basis of three main APA, namely, ACL, LAC, and anti-*β*_2_GPI. Although we did not identify loci meeting the conservative threshold of genome-wide significance, we have identified a number of suggestive novel loci for APA that will stimulate follow-up studies in independent and larger sample sets to replicate our findings. The main limitations of our study include relatively small sample size and lack of a replication sample; however, our top SNPs provide a select group of suggestive candidate loci/genes that can easily be tested for replication by other research groups, which would also enable a subsequent meta-analysis with increased power.

## Figures and Tables

**Table 1:** Characteristics of study participants with three antiphospholipid antibodies in the GWAS dataset*.

	ACL		LAC		Anti-*β*_2_GPI	
	Positive	Negative	Positive	Negative	Positive	Negative
	(*n* = 183)	(*n* = 487)	(*n* = 127)	(*n* = 581)	(*n* = 136)	(*n* = 360)
Mean age ± SD	46.92 ± 11.41	46.19 ± 10.85	45.64 ± 11.35	46.40 ± 11.36	45.91 ± 10.68	46.79 ± 11.18
SLE cases (%)	58.5	58.1	70.8	56.3	71.3	51.9
Controls (%)	41.5	41.9	29.2	43.7	28.7	48.1

*ACL: anticardiolipin antibodies; LAC: lupus anticoagulant; Anti-*β*_2_GPI: anti-*β*_2_ glycoprotein I antibodies.

**Table 2:** Genetic loci associated with the occurrence of ACL with *P* < 1*E* − 04*.

CHR	Gene	Lead SNP	BP	Total SNPs	MAF		OR	*P*
					Negative	Positive		
5	*PELO *	rs6889746	51742663	3	0.3534	0.5	1.776	6.02*E* − 06
1	*SGIP1 *	rs6681460	66895645	28	0.3755	0.5168	1.827	6.98*E* − 06
6	*LCA5 *	rs12204683	80212978	4	0.2378	0.3701	1.877	7.02*E* − 06
4	*MIR4275 *	rs17642174	28160435	6	0.1355	0.2346	2.021	1.42*E* − 05
4	*C4orf37 *	rs13134014	99323902	21	0.1517	0.2542	1.928	1.76*E* − 05
7	*BZW2 *	rs6961256	16697717	3	0.01261	0.05587	5.214	1.96*E* − 05
2	*FAM49A *	rs6753768	16483918	11	0.2836	0.1648	0.5016	2.37*E* − 05
4	*MAD2L1 *	rs10518344	1.21*E* + 08	3	0.05136	0.1178	2.696	2.77*E* − 05
11	*KIRREL3 *	rs1793667	1.26*E* + 08	1	0.2789	0.3966	1.781	2.89*E* − 05
14	*YLPM1 *	rs2241275	74321193	7	0.435	0.5587	1.754	3.06*E* − 05
14	*PROX2 *	rs4899536	74386367	2	0.4331	0.5559	1.752	3.28*E* − 05
2	*ATL2 *	rs6749177	38531205	11	0.4958	0.3659	0.576	3.77*E* − 05
17	*PRPSAP1 *	rs11077813	71848140	1	0.2704	0.1564	0.5072	3.82*E* − 05
9	*TTLL11 *	rs10985483	1.24*E* + 08	4	0.3703	0.25	0.5571	4.09*E* − 05
8	*ZBTB10 *	rs406629	81620942	6	0.3795	0.2599	0.5501	4.26*E* − 05
21	*LINC00317 *	rs2827107	22175926	3	0.2128	0.3156	1.847	5.63*E* − 05
7	*NUPL2 *	rs10232205	23197079	1	0.09244	0.02793	0.2465	6.22*E* − 05
1	*DUSP10 *	rs11118750	2.2*E* + 08	4	0.2174	0.3287	1.753	6.88*E* − 05
15	*MCTP2 *	rs1863095	92917093	1	0.3224	0.4511	1.665	6.90*E* − 05
17	*SHPK *	rs222790	3478239	1	0.2051	0.3097	1.793	7.30*E* − 05
6	*KHDRBS2 *	rs2752976	63486388	11	0.431	0.5447	1.688	7.88*E* − 05
5	*GLRA1 *	rs154111	1.51*E* + 08	14	0.4569	0.3399	0.5875	7.91*E* − 05
9	*MIR548AA1 *	rs4836873	1.24*E* + 08	4	0.3651	0.2514	0.5746	8.29*E* − 05
1	*LOC100505918 *	rs16860501	1.67*E* + 08	1	0.09119	0.1648	2.116	8.89*E* − 05
19	*OR7A10 *	rs4808564	14825095	1	0.05126	0.1117	2.449	8.91*E* − 05
9	*C9orf46 *	rs4742085	5340548	2	0.3501	0.4678	1.696	9.02*E* − 05
9	*PTPRD *	rs2484741	10477877	7	0.2977	0.1983	0.5439	9.79*E* − 05
16	*PDXDC1 *	rs3198697	15037441	1	0.3658	0.4859	1.646	9.83*E* − 05

*CHR: chromosome; Gene: a plausible biological candidate gene in the locus or the nearest annotated gene to the lead SNP; Lead SNP: most significant SNP in the gene region; BP: base-pair position of the lead SNP; Total SNPs: total number of SNPs with *P* < 1*E* − 03 in the gene region; MAF: minor allele frequencies in antibody-negative and antibody-positive groups; OR: odds ratio; *P*: *P*-values for the test.

**Table 3:** Genetic loci associated with the occurrence of lupus anticoagulant (LAC) with *P* < 1*E* − 04*.

CHR	Gene	Lead SNP	BP	Total SNPs	MAF		OR	*P*
					Negative	Positive		
22	*MICAL3 *	rs1978968	16828113	8	0.2144	0.3553	2.235	2.21*E* − 06
2	*FAM176A *	rs17011455	75643997	1	0.01661	0.06967	5.211	4.70*E* − 06
20	*DSTN *	rs17791782	17514069	2	0.07867	0.1721	2.628	6.54*E* − 06
6	*SUPT3H *	rs9472374	44904278	1	0.01957	0.07083	4.77	1.14*E* − 05
3	*LRIG1 *	rs4549225	66850149	10	0.3735	0.5246	1.867	1.54*E* − 05
1	*SMYD3 *	rs7527610	2.45*E* + 08	2	0.005236	0.04098	10.19	1.77*E* − 05
14	*PELI2 *	rs754314	55822350	8	0.04833	0.1167	3.111	2.13*E* − 05
20	*BFSP1 *	rs16999416	17489830	1	0.08494	0.1777	2.412	2.32*E* − 05
16	*NDRG4 *	rs11862356	57067820	1	0.05846	0.1393	2.745	2.40*E* − 05
17	*CDRT15P1 *	rs7208809	13678048	2	0.06806	0.1516	2.573	2.62*E* − 05
22	*FAM19A5 *	rs9615320	47219591	2	0.1065	0.2008	2.333	3.07*E* − 05
6	*SNRNP48 *	rs17398435	7549105	1	0.06392	0.1446	2.589	3.45*E* − 05
14	*OTX2 *	rs12897597	56314078	1	0.1531	0.2667	2.097	3.53*E* − 05
17	*RBFOX3 *	rs16972153	74719776	1	0.04974	0.1148	2.925	3.59*E* − 05
16	*MAF *	rs9935211	78440577	5	0.05467	0.1311	2.692	4.18*E* − 05
5	*YTHDC2 *	rs6865651	1.13*E* + 08	2	0.1848	0.3058	2.005	4.32*E* − 05
10	*LDB3 *	rs4934256	88490345	2	0.05507	0.123	2.783	4.68*E* − 05
9	*KLF4 *	rs1888617	1.1*E* + 08	1	0.2657	0.4009	1.935	4.92*E* − 05
10	*TACC2 *	rs12773310	1.24*E* + 08	6	0.3536	0.219	0.4945	5.19*E* − 05
6	*LY86 *	rs9328374	6536628	1	0.1875	0.307	1.989	5.40*E* − 05
9	*ZCCHC7 *	rs7031314	37366122	3	0.2204	0.3375	1.924	5.90*E* − 05
3	*SETD5 *	rs17050346	9456593	5	0.01926	0.06967	4.047	6.34*E* − 05
4	*COL25A1 *	rs13104799	1.1*E* + 08	6	0.2245	0.1107	0.4106	6.68*E* − 05
7	*C7orf58 *	rs12537243	1.2*E* + 08	1	0.09178	0.1736	2.289	7.22*E* − 05
4	*RBM46 *	rs7687314	1.56*E* + 08	6	0.4474	0.5902	1.808	7.25*E* − 05
9	*LOC100506710 *	rs10973184	37056617	1	0.09895	0.1885	2.252	7.33*E* − 05
3	*EPHA6 *	rs4318565	97564200	1	0.03369	0.08607	3.32	7.77*E* − 05
11	*LOC283143 *	rs1393275	1.15*E* + 08	3	0.05026	0.1261	2.595	7.90*E* − 05
18	*MAPRE2 *	rs573269	30863716	1	0.3129	0.4385	1.816	8.05*E* − 05
13	*FARP1 *	rs285031	97584032	1	0.1489	0.2438	2.092	8.11*E* − 05
10	*ANXA2P3 *	rs10822492	66751936	15	0.3439	0.219	0.5075	8.32*E* − 05
6	*TBCC *	rs11759402	42831787	4	0.1658	0.2686	1.945	8.32*E* − 05
4	*LNX1 *	rs6831173	54085908	4	0.1337	0.2377	1.962	8.80*E* − 05
4	*SLC7A11 *	rs10440463	1.39*E* + 08	3	0.2073	0.3238	1.88	8.85*E* − 05
1	*HHAT *	rs1028383	2.09*E* + 08	1	0.2248	0.3475	1.882	9.29*E* − 05
11	*WT1 *	rs2207549	32325033	2	0.4202	0.2833	0.5274	9.37*E* − 05
4	*FAM198B *	rs17036867	1.59*E* + 08	12	0.02747	0.08621	3.54	9.54*E* − 05

*CHR: chromosome; Gene: a plausible biological candidate gene in the locus or the nearest annotated gene to the lead SNP; Lead SNP: most significant SNP in the gene region; BP: base-pair position of the lead SNP; Total SNPs: total number of SNPs with *P* < 1*E* − 03 in the gene region; MAF: minor allele frequencies in antibody-negative and antibody-positive groups; OR: odds ratio; *P*: *P*-values for the test.

**Table 4:** Genetic loci associated with the occurrence of anti-*β*_2_GPI antibodies with *P* < 1*E* − 04*.

CHR	Gene	Lead SNP	BP	Total SNPs	MAF		OR	*P*
					Negative	Positive		
13	*MYO16 *	rs10492418	108178727	5	0.4292	0.6016	2.172	2.05*E* − 06
7	*PDE1C *	rs11975235	32156065	4	0.4843	0.311	0.4675	2.88*E* − 06
2	*TANK *	rs2357982	161594349	13	0.2359	0.3885	2.189	3.38*E* − 06
13	*FLJ42392 *	rs9315762	39639907	2	0.1535	0.2901	2.255	6.68*E* − 06
20	*MACROD2 *	rs6080100	15951406	7	0.2655	0.4275	2.086	6.86*E* − 06
17	*CAMKK1 *	rs758642	3733656	1	0.3314	0.4837	2.038	1.23*E* − 05
14	*ITPK1 *	rs8021497	92637371	6	0.2059	0.3508	2.101	1.26*E* − 05
2	*SESTD1 *	rs10186547	179921914	2	0.3853	0.5391	2.009	1.56*E* − 05
10	*CACNB2 *	rs12356676	18687510	6	0.2587	0.3943	2.134	1.80*E* − 05
18	*LRRC30 *	rs9965173	7201755	4	0.07887	0.1718	2.647	2.59*E* − 05
3	*PEX5L *	rs9856007	181225431	3	0.2514	0.3817	1.976	3.68*E* − 05
11	*TMEM45B *	rs10894119	129081436	1	0.1835	0.293	2.168	4.05*E* − 05
10	*SFTA1P *	rs1000039	10597879	1	0.02254	0.08779	4.19	4.13*E* − 05
9	*OR1J1 *	rs2778636	124270913	2	0.2641	0.3931	1.955	4.43*E* − 05
5	*RAPGEF6 *	rs17671387	130911895	1	0.04507	0.1183	3.186	4.71*E* − 05
2	*GMCL1 *	rs4241261	69917164	3	0.4761	0.3308	0.5278	4.93*E* − 05
14	*C14orf101 *	rs7153196	56256660	2	0.362	0.229	0.4996	5.58*E* − 05
9	*WNK2 *	rs10821084	94991443	1	0.07627	0.1718	2.425	6.33*E* − 05
10	*SLC16A9 *	rs7082987	61007793	1	0.3663	0.2317	0.4886	6.46*E* − 05
4	*GDEP *	rs11730315	81020089	2	0.1648	0.2829	2.075	7.24*E* − 05
4	*ARHGAP24 *	rs17010960	86938938	2	0.02254	0.07634	4.326	7.85*E* − 05
13	*HTR2A *	rs582385	46343995	4	0.1624	0.2824	2.02	8.09*E* − 05
15	*FMN1 *	rs2444955	31199305	2	0.2211	0.355	1.974	8.10*E* − 05
5	*CARTPT *	rs16869487	70976850	1	0.02841	0.09542	3.602	8.59*E* − 05
2	*NEU2 *	rs11695991	233608333	1	0.0169	0.0687	4.802	8.63*E* − 05
17	*CA10 *	rs203076	47354484	7	0.2944	0.1603	0.4729	8.70*E* − 05
9	*C9orf135 *	rs1389124	71669176	3	0.09943	0.1985	2.286	9.23*E* − 05
20	*SMOX *	rs1764996	4070805	2	0.02817	0.07634	4.014	9.93*E* − 05

*CHR: chromosome; Gene: a plausible biological candidate gene in the locus or the nearest annotated gene to the lead SNP; Lead SNP: most significant SNP in the gene region; BP: base-pair position of the lead SNP; Total SNPs: total number of SNPs with *P* < 1*E* − 03 in the gene region; MAF: minor allele frequencies in antibody-negative and antibody-positive groups; OR: odds ratio; *P*: *P*-values for the test.

**Table 5:** Genetic loci associated with the occurrence of two or more antiphospholipid antibodies (ACL, LAC, or Anti-*β*_2_GPI) with *P* < 1*E* − 04*.

CHR	Gene	Lead SNP	BP	Total SNPs	MAF		OR	*P*
					Negative	Positive		
16	*DYNLRB2 *	rs8060581	78750106	6	0.02466	0.1406	6.714	1.44*E* − 06
2	*SESTD1 *	rs13403289	179924976	2	0.3857	0.5833	2.423	6.08*E* − 06
1	*DNAH14 *	rs3913653	223603694	11	0.543	0.3421	0.43	1.09*E* − 05
10	*CACNB2 *	rs10828616	18710023	6	0.2175	0.3698	2.538	1.11*E* − 05
18	*EPB41L3 *	rs7238186	5469093	2	0.213	0.3854	2.327	2.61*E* − 05
1	*MAGI3 *	rs11102625	113750976	5	0.4355	0.6146	2.298	2.75*E* − 05
2	*TANK *	rs13010671	161593338	5	0.07442	0.1882	3.437	3.17*E* − 05
7	*CNTNAP2 *	rs12113442	145329124	1	0.1054	0.2188	2.938	3.43*E* − 05
18	*ZNF519 *	rs8093228	13989380	4	0.2152	0.3646	2.326	4.85*E* − 05
3	*FAM198A *	rs7624799	43020807	3	0.1592	0.2969	2.466	5.00*E* − 05
20	*DSTN *	rs17791782	17514069	2	0.07883	0.1927	3.086	5.06*E* − 05
20	*BFSP1 *	rs16999416	17489830	1	0.08371	0.2031	2.93	5.63*E* − 05
13	*ANKRD20A9P *	rs7319595	18392986	1	0.3484	0.5213	2.337	5.75*E* − 05
15	*TLE3 *	rs10518889	68337482	4	0.2838	0.4427	2.231	6.17*E* − 05
22	*CLTCL1 *	rs8135222	17672446	1	0.1749	0.3281	2.293	6.19*E* − 05
11	*TMEM45B *	rs10894119	129081436	1	0.1789	0.3158	2.432	6.25*E* − 05
1	*GADD45A *	rs787480	67868460	1	0.2207	0.08854	0.3088	6.57*E* − 05
20	*SPTLC3 *	rs6105044	13065747	2	0.352	0.1979	0.4138	7.08*E* − 05
6	*HIVEP1 *	rs6908010	12325985	2	0.4753	0.3073	0.4688	7.11*E* − 05
7	*CUX1 *	rs427534	101673424	3	0.4439	0.2656	0.4569	7.28*E* − 05
10	*MSMB *	rs7094791	51229942	1	0.3857	0.2396	0.4081	7.35*E* − 05
8	*TPD52 *	rs10090469	81396522	1	0.1099	0.2292	2.668	7.43*E* − 05
14	*LOC100506433 *	rs698322	47597316	1	0.3914	0.2344	0.4277	7.58*E* − 05
6	*MMS22L *	rs1206164	97703068	1	0.2511	0.4219	2.212	8.26*E* − 05
12	*CDK17 *	rs11108526	95379901	2	0.02691	0.08333	5.95	8.40*E* − 05
11	*PDGFD *	rs4754095	103278377	3	0.2851	0.1436	0.3771	8.83*E* − 05
15	*FMN1 *	rs2444955	31199305	2	0.1951	0.3474	2.408	8.89*E* − 05
10	*SORCS1 *	rs4918273	108715485	11	0.3597	0.5319	2.064	8.92*E* − 05

*CHR: chromosome; Gene: a plausible biological candidate gene in the locus or the nearest annotated gene to the lead SNP; Lead SNP: most significant SNP in the gene region; BP: base-pair position of the lead SNP; Total SNPs: total number of SNPs with *P* < 1*E* − 03 in the gene region; MAF: minor allele frequencies in antibody-negative (negative for ALC, ACL and anti-*β*_2_GPI) and antibody-positive (positive for at least two of ALC, ACL or anti-*β*_2_GPI) groups; OR: odds ratio; *P*: *P*-values for the test.

**Table 6:** Significant SNPs with *P* < 0.01 in the MHC region on chromosome 6 for ACL, LAC, and Anti-*β*_2_GP1*.

Gene	SNP	*P *
ACL		

*HLA-DPB1 *	rs3128918	0.00028
*HLA-DPB2 *	rs2395357	0.00043
*HLA-DMA *	rs11539216	0.00099
*HLA-DQB2 *	rs10484564	0.00536
*GNL1 *	rs9295888	0.00758
*GNL1 *	rs9295873	0.00794
*HLA-DOA *	rs4713603	0.0081
*RPP21 *	rs1548515	0.00842
*GNL1 *	rs9461607	0.00863
*GNL1 *	rs17411480	0.00863
*RPP21 *	rs9261821	0.00863
*RPP21 *	rs9261850	0.00863
*RPP21 *	rs9261854	0.00863
*RPP21 *	rs9261855	0.00863
*RPP21 *	rs1548513	0.00863
*RPP21 *	rs9261925	0.00863
*RPP21 *	rs9261926	0.00863
*BRD2 *	rs17840186	0.00939
*RPP21 *	rs9261799	0.00955

LAC		

*TAP2 *	rs1044043	0.00029
*HLA-DQA1 *	rs642093	0.00032
*AIF1 *	rs2736177	0.00041
*HLA-DQA2 *	rs9275765	0.00078
*HLA-DQA2 *	rs9275772	0.00078
*HLA-DQA2 *	rs9275793	0.00088
*HLA-DQA1 *	rs9272346	0.00130
*HLA-DQA2 *	rs9276298	0.00133
*HLA-DQA1 *	rs9272219	0.00167
*C6orf10 *	rs3129934	0.00168
*HLA-DQA1 *	rs9272535	0.00179
*HLA-DRB1 *	rs674313	0.00215
*HLA-DRB1 *	rs502771	0.00227
*HLA-DRB1 *	rs9270986	0.00250
*AIF1 *	rs2857597	0.00257
*HCG26 *	rs2516516	0.00282
*HLA-DRB1 *	rs615672	0.00295
*HLA-DRB1 *	rs502055	0.00300
*HLA-DQA1 *	rs9272723	0.00329
*LOC100294145 *	rs9276915	0.00352
*C6orf10 *	rs2894254	0.00372
*UBD *	rs9368606	0.00403
*C6orf10 *	rs3129900	0.00458
*MCCD1 *	rs2734573	0.00553
*PRRC2A *	rs1046080	0.00618
*C6orf10 *	rs7767325	0.00623
*C6orf15 *	rs2517448	0.00627
*C6orf10 *	rs3132928	0.00640
*HLA-H *	rs3132722	0.00675
*HLA-DQB2 *	rs2857210	0.00685
*HLA-DRA *	rs3129868	0.00731
*ATP6V1G2-DDX39B/DDX39B *	rs933208	0.00740
*NFKBIL1 *	rs2857605	0.00755
*TRIM26 *	rs3132671	0.00757
*HLA-DQB1 *	rs3129716	0.00770
*MSH5/MSH5-C6orf26 *	rs3131379	0.00849
*MSH5/MSH5-C6orf26 *	rs3130484	0.00901
*PSMB9 *	rs9276832	0.00907
*BTNL2 *	rs2213581	0.00922
*HLA-DMA *	rs11539216	0.00929
*ATP6V1G2-DDX39B/DDX39B *	rs3093978	0.00957
*C6orf10 *	rs2143461	0.00986
*TUBB *	rs3095330	0.00990
*LOC100294145 *	rs4959119	0.00992
*TRIM26 *	rs2517611	0.00993

Anti-*β*_2_GPI		

*HLA-DPB2 *	rs9277916	0.00147
*HLA-DQA2 *	rs9275793	0.00310
*HLA-DQA2 *	rs9275765	0.00315
*HLA-DQA2 *	rs9275772	0.00315
*HLA-DPA1 *	rs3130182	0.00331
*HLA-DQB1 *	rs9469220	0.00372
*HLA-DPB2 *	rs4711314	0.00378
*HLA-DQA2 *	rs2647089	0.00463
*HLA-DQA2 *	rs9276298	0.00523
*HLA-DQB1 *	rs9275356	0.00526
*HLA-DQA2 *	rs17615250	0.00710
*HLA-DQA2 *	rs9275618	0.00807

*ACL: anticardiolipin antibodies; LAC: lupus anticoagulant; Anti-*β*_2_GPI: anti-*β*_2_ glycoprotein I antibodies; Gene: a plausible biological candidate gene in the locus or the nearest annotated gene to the SNP; SNP: single-nucleotide polymorphism; *P*: *P*-values for the test.

**Table 7:** Odds ratios and *P*-values for the association analysis of *APOH* SNPs on chromosome 17 with ACL, LAC, and anti-*β*_2_GPI*.

SNP	BP	ACL		LAC		Anti-*β*_2_GPI	
		OR	*P*	OR	*P*	OR	*P*
rs1801690	61638747	0.7655	0.4048	0.6444	0.2614	2.461	0.003122
rs17769836	61663751	0.8968	0.4457	1.004	0.9821	0.5978	0.004849
rs2873966	61642435	0.9519	0.7168	0.9858	0.9262	0.6224	0.005407
rs7215391	61662484	0.8932	0.4391	0.984	0.9227	0.6146	0.008069
rs8073418	61678134	0.9639	0.7759	0.8949	0.455	0.6661	0.01061
rs8064837	61673165	0.9833	0.8915	1.011	0.9388	1.404	0.02235
rs10491174	61685021	1.017	0.9335	0.765	0.2665	1.487	0.07256
rs2215413	61679959	0.9418	0.6397	0.842	0.2435	0.829	0.221
rs16958979	61654321	0.8586	0.5569	0.4332	0.02739	1.375	0.2253
rs8178822	61655991	0.8748	0.6127	0.407	0.0258	1.379	0.2345
rs12452959	61635526	0.8991	0.5748	1.196	0.3965	1.275	0.2541
rs4791079	61640002	1.128	0.3573	1.206	0.2028	1.181	0.2802
rs3176975	61641219	0.9723	0.8552	0.9923	0.9649	1.209	0.2858
rs8066294	61673500	1.094	0.5597	1.014	0.9365	1.162	0.3981
rs17763430	61635203	0.998	0.9904	0.8987	0.5811	0.8448	0.4051
rs17690171	61633319	0.9893	0.9435	1.172	0.3479	1.141	0.4528
rs16959003	61671199	1.105	0.6095	0.8378	0.4548	1.14	0.5639
rs735866	61670208	1.206	0.3459	0.8419	0.4765	1.117	0.642
rs7208089	61689374	1.091	0.5465	0.7956	0.1906	1.08	0.6476
rs7222710	61630703	1.089	0.5011	1.016	0.9143	0.942	0.6896
rs6933	61638692	1.126	0.3408	1.068	0.6431	1.02	0.8948

*APOH: apolipoprotein H; SNP: single-nucleotide polymorphism; BP: base-pair position; OR: odds ratio; *P*: *P*-values. ACL: anticardiolipin antibodies; LAC: lupus anticoagulant; Anti-*β*_2_GPI: anti-*β*_2_ glycoprotein I antibodies.

## References

[1] Gharavi AE, Wilson WA, Wallace DJ, Hahn BH (1997). Antphospholipid antibodies. *Dubois'Lupus Erythematosus*.

[2] Roubey RAS (2000). Update on antiphospholipid antibodies. *Current Opinion in Rheumatology*.

[3] Pierangeli SS, De Groot PG, Dlott J (2011). “Criteria” aPL tests: report of a task force and preconference workshop at the 13th International Congress on Antiphospholipid Antibodies, Galveston, Texas, April 2010. *Lupus*.

[4] Mackworth-Young C, Chan J, Harris N (1987). High incidence of anticardiolipin antibodies in relatives of patients with systemic lupus erythematosus. *Journal of Rheumatology*.

[5] Matthey F, Walshe K, Mackie IJ, Machin SJ (1989). Familial occurrence of the antiphospholipid syndrome. *Journal of Clinical Pathology*.

[6] Horita T, Merrill JT (2004). Genetics of antiphospholipid syndrome. *Current rheumatology reports*.

[7] Png E, Alisjahbana B, Sahiratmadja E (2012). A genome wide association study of pulmonary tuberculosis susceptibility in Indonesians. *BMC Medical Genetics*.

[8] Miehe S, Bieberstein A, Arnould I, Ihdene O, Rütten H, Strübing C (2010). The phospholipid-binding protein SESTD1 is a novel regulator of the transient receptor potential channels TRPC4 and TRPC5. *Journal of Biological Chemistry*.

[9] Shitao L, Lingyan W, Michael B (2011). Mapping of dynamic innate immunity protein interaction network regulating type I interferon production. *Immunity*.

[10] Song QL, He XX, Yang H (2012). Association of TANK gene polymorphism with outcomes of hepatitis B virus infection in a Chinese Han population. *Viral Immunology*.

[11] Mosbruger TL, Duggal P, Goedert JJ (2010). Large-scale candidate gene analysis of spontaneous clearance of hepatitis C virus. *Journal of Infectious Diseases*.

[12] Pezzolesi MG, Poznik GD, Mychaleckyj JC (2008). Variants in KCNQ1 are associated with susceptibility to type 2 diabetes mellitus. *Nature Genetics*.

[13] Maeda S, Araki SI, Babazono T (2010). Replication study for the association between four loci identified by a genome-wide association study on European American subjects with type 1 diabetes and susceptibility to diabetic nephropathy in Japanese subjects with type 2 diabetes. *Diabetes*.

[14] Pezzolesi MG, Poznik GD, Skupien J (2011). An intergenic region on chromosome 13q33.3 is associated with the susceptibility to kidney disease in type 1 and 2 diabetes. *Kidney International*.

[15] Gigante A, Gasperini ML, Cianci R (2009). Antiphospholipid antibodies and renal involvement. *American Journal of Nephrology*.

[16] Tektonidou MG (2009). Renal involvement in the antiphospholipid syndrome (APS) - APS nephropathy. *Clinical Reviews in Allergy and Immunology*.

[17] Silvarino R, Sant F, Espinosa G (2011). Nephropathy associated with antiphospholipid antibodies in patients with systemic lupus erythematosus. *Lupus*.

[18] Mehrani T, Petri M (2011). IgM anti-*β*
_2_ glycoprotein I is protective against lupus nephritis and renal damage in systemic lupus erythematosus. *Journal of Rheumatology*.

[19] Wang L, Yu C, Lu Y (2007). TMEM166, a novel transmembrane protein, regulates cell autophagy and apoptosis. *Apoptosis*.

[20] Pierdominici M, Vomero M, Barbati C (2012). Role of autophagy in immunity and autoimmunity, with a special focus on systemic lupus erythematosus. *FASEB Journal*.

[21] Mũoz LE, Lauber K, Schiller M, Manfredi AA, Herrmann M (2010). The role of defective clearance of apoptotic cells in systemic autoimmunity. *Nature Reviews Rheumatology*.

[22] Chamorro A-J, Marcos M, Miron-Canelo J-A (2012). Val247Leu beta2-glycoprotein-I allelic variant is associated with antiphospholipid syndrome: systemic review and meta-analysis. *Autoimmunity Reviews*.

[23] Sanghera DK, Wagenknecht DR, McIntyre JA, Kamboh MI (1997). Identification of structural mutations in the fifth domain of apolipoprotein H (*β*
_2_-glycoprotein I) which affect phospholipid binding. *Human Molecular Genetics*.

